# FLEXIBILITY MEETS COMPLEXITY: LESSONS FROM EMBEDDING A DEMENTIA CARE COORDINATION MODEL IN PRACTICE

**DOI:** 10.1093/geroni/igac059.1284

**Published:** 2022-12-20

**Authors:** Quincy Samus

**Affiliations:** Johns Hopkins School of Medicine, Baltimore, Maryland, United States

## Abstract

People living with dementia (PLWD) are among the highest-need and highest-cost individuals because of the complexity, duration, and range of medical, behavioral, environmental, and social needs. There is a growing evidence base showing that family-centered active management approaches that include activation and empowerment of care partners are well suited to improve care quality and health-related outcomes, and have potential to curb high ADRD-related healthcare costs. This presentation will outline key experiences and lessons learned after a decade of work developing, adapting and embedding a comprehensive family-focused care management model called MIND at Home into practice. The work, supported in part by the IMPACT Collaboratory Health Care System Scholars Award to partner with Centene Corporation, a large managed care organization, illustrates two overriding principles: (1) the necessity of “meeting people and health systems where they are” (literally and figuratively), and (2) the importance of effectively matching intervention to outcome and context.

